# Mechanical Force Promotes Mitochondrial Transfer From Macrophages to BMSCs to Enhance Bone Formation

**DOI:** 10.1111/cpr.70121

**Published:** 2025-08-27

**Authors:** Yingyi Li, Ziwei Yan, Yueming Dai, Hanjia Cai, Yue Chen, Yuyi Chen, Ruofan Jin, Wen Sun, Hua Wang

**Affiliations:** ^1^ Department of Orthodontics The Affiliated Stomatological Hospital of Nanjing Medical University Nanjing China; ^2^ Department of Basic Science of Stomatology The Affiliated Stomatological Hospital of Nanjing Medical University Nanjing China; ^3^ State Key Laboratory Cultivation Base of Research Prevention and Treatment for Oral Diseases Nanjing China; ^4^ Jiangsu Province Engineering Research Center of Stomatological Translational Medicine Nanjing China

**Keywords:** bone marrow mesenchymal stem cell, macrophage, mechanical tension, mitochondria transfer, osteogenesis

## Abstract

Macrophages and bone marrow mesenchymal stem cells (BMSCs) share a close relationship within the osteoimmune microenvironment. During mechanically induced bone formation, macrophages respond to stimuli and regulate this microenvironment, influencing BMSCs' proliferation and differentiation. However, the underlying mechanisms remain incompletely understood. In our study, we employed a cellular tension system and found that mechanical tension altered mitochondrial dynamics in macrophages, leading to increased mitochondrial fission. Using a macrophage‐BMSC direct co‐culture system, we demonstrated that macrophages transferred mitochondria to BMSCs, a process enhanced by tension. This enhancement was associated with Drp1‐mediated mitochondrial fission, as Drp1 knockdown in macrophages abolished the effect. Additionally, using in vitro co‐culture and in vivo tibial injection models, we found that mitochondria‐rich extracellular vesicles (Mito‐EVs) secreted by mechanically stretched macrophages promoted BMSCs' osteogenesis and enhanced bone formation via the CD200 receptor (CD200R)‐CD200 interaction. Our findings reveal a pivotal role for mitochondrial transfer in promoting osteogenesis during mechanotransduction, highlighting a novel mechanism of intercellular communication in bone biology.

## Graphical abstract

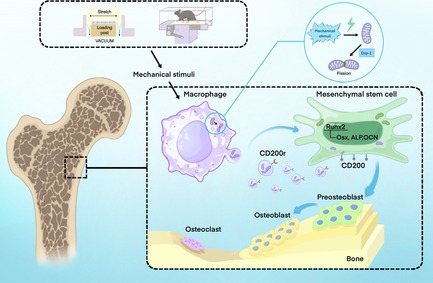

## 1 Introduction

Mechanical stimuli are closely associated with various essential life processes and are indispensable for maintaining biological functions [1]. Mechanosensitive cells recognise mechanical stimuli and convert these signals—such as compression [2], tension [3] or shear stress [4]—into biochemical signals, leading to adaptive functional changes. This process is known as mechanotransduction. Bone is highly metabolically active and continuously undergoes resorption and remodelling [5]. Favourable mechanical forces contribute to maintaining bone homeostasis and regulating bone formation, as observed in processes like physical exercise, distraction osteogenesis and orthodontic tooth movement [6, 7, 8]. Therefore, investigating the mechanisms of mechanotransduction is of great significance for the field of bone regeneration medicine.

The concept of the osteoimmune microenvironment has gained increasing attention in recent years [9, 10]. Osteogenic lineage cells and immune cells coexist within the bone marrow microenvironment, where the skeletal and immune systems are closely interconnected and mutually influence one another [11]. Macrophages, derived from haematopoietic stem cells (HSCs) in the bone marrow, are essential immune cells with diverse functions in host defence, tissue homeostasis and repair [12]. Bone marrow mesenchymal stem cells (BMSCs) are self‐renewal and multipotent stem cells capable of differentiating into various cell types including osteoblasts, which are essential for bone formation [13]. Extensive research has demonstrated that macrophages and BMSCs engage in crosstalk during osteogenesis, primarily through macrophage‐secreted cytokines, chemokines or microRNAs, activating downstream osteogenic signalling pathways [14, 15, 16]. Under mechanical forces, in addition to the response of bone cells [7, 17, 18, 19], macrophages also exhibit mechanosensitivity, which in turn regulates the osteogenic differentiation by influencing the osteoimmune microenvironment [20, 21, 22]. However, the mechanisms underlying this crosstalk remain largely unexplored, considering the complexity and diversity of cell‐to‐cell communication.

Recently, a novel form of intercellular communication—mitochondrial transfer—has garnered increasing attention [23]. The migration of mitochondria from donor to recipient cells profoundly affects recipient cell function, typically beneficial, such as maintaining tissue homeostasis under physiological conditions [24] or restoring function in damaged cells under pathological conditions [25, 26]. However, it can also have adverse effects, such as promoting tumour cell invasion and proliferation [27]. Mitochondria, known as the cell's ‘powerhouse’, produce ATP through mitochondrial respiration and oxidative phosphorylation, thereby supporting cellular energy metabolism [28]. They also play key roles in cell differentiation [29], signal transduction [30] and apoptosis [31], which are closely linked to bone metabolism. Consistently, it was reported that artificial mitochondrial transfer can significantly enhance the proliferation, migration and osteogenic potential of recipient BMSCs [32].

Mitochondrial dynamics involve the continual regulation of mitochondrial morphology and quantity through fusion and fission processes [33]. Mitochondrial fusion is primarily mediated by Mitofusin 1 (Mfn1), Mitofusin 2 (Mfn2) and Optic Atrophy 1 (Opa1), while fission is regulated by Dynamin‐related protein 1 (Drp1) and Fission 1 (Fis1) [33]. Mitochondrial dynamics are closely associated with cellular functions. For example, enhanced mitochondrial dynamics have been shown to promote spermatogonial differentiation [34]. Moreover, studies have found that mitochondrial fission in osteoblasts facilitates osteogenesis [35]. In terms of mechanical force and mitochondrial dynamics, some studies suggest that mitochondrial dynamics mediate mechanotransduction [36]. In pulmonary fibroblasts and endothelial cells, mechanical forces have been shown to alter mitochondrial morphology, increase intracellular ROS levels and influence cellular functions [37, 38]. However, there has been few similar research in macrophages.

Notably, a recent study reported mitochondrial transfer from macrophages to BMSCs, indicating mitochondria‐mediated communication between these cell types [39]. Current evidence suggests that mitochondrial transfer occurs via tunnelling nanotubes (TNTs) [40], as well as through the secretion of microvesicles and free mitochondria [41]. In addition to the release of intact mitochondria, cells are capable of releasing mitochondrial DNA fragments in specific contexts, for example, in the form of mitochondrial neutrophils extracellular traps (mtNETs) [42]. However, the mechanisms regulating mitochondrial transfer between specific cell types, the way recipient cells internalise mitochondria and the potential to enhance such transfer remain largely unknown.

In this study, we hypothesised that mechanical tension could regulate macrophage mitochondrial function, thereby influencing mitochondrial communication with BMSCs and modulating their osteogenic differentiation. We applied cyclic tension to RAW264.7, a murine macrophage cell line, to mimic the in vivo mechanical loading environment. Mitochondria‐rich extracellular vesicles (Mito‐EVs) isolated from macrophages were injected into mice to verify the role of mitochondrial transfer in bone formation. Our findings indicate that mechanical tension activates Drp1 and induces mitochondrial fission, promotes the transfer of macrophage mitochondria to BMSCs, thus enhancing BMSCs osteogenic differentiation and in vivo bone formation. Additionally, we identified that the CD200 receptor (CD200R)‐CD200 interaction may facilitate the recognition and internalisation of macrophage‐derived Mito‐EVs by BMSCs.

## 2 Results

### 2.1 Running‐Induced Bone Mass Increase in Mice May Be Associated With Mitochondrial Transfer From Macrophages to BMSCs

**FIGURE 1:**
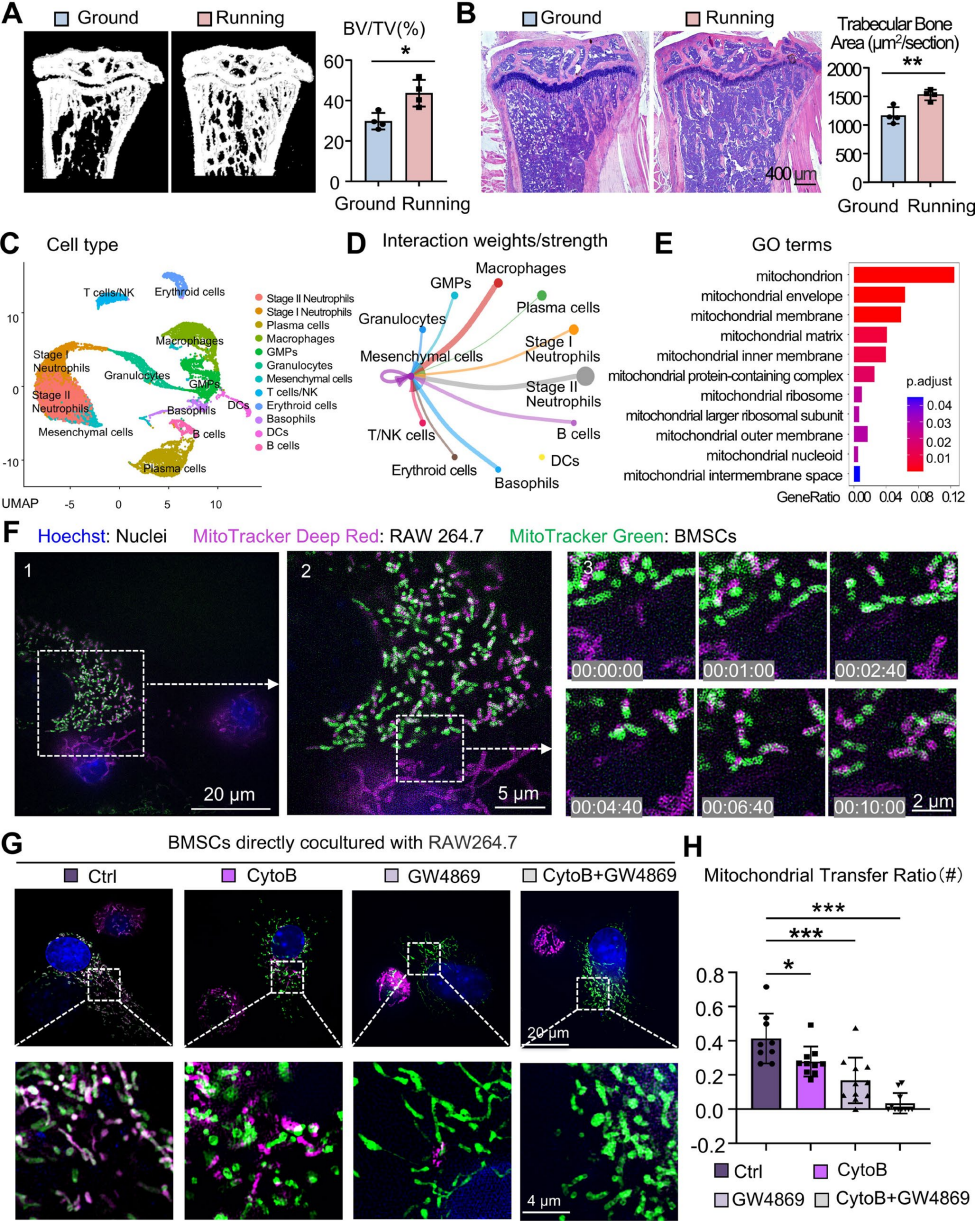
Running‐induced bone mass increase in mice may be associated with mitochondrial transfer from macrophages to BMSCs. (A, B) Eight‐week‐old mice were subjected to a mechanical loading exercise regimen as described in Methods (Running group). Littermate mice that did not perform the exercise served as controls (Ground group). After 1 month, the mice were sacrificed for analysis. *N* = 4 per group. (A) Representative images of three‐dimensional micro‐CT reconstructions of tibiae. Bone volume/total volume (BV/TV) (%) was determined. (B) Representative HE‐stained paraffin sections of tibiae. Trabecular bone area (μm^2^/field) was determined. (C, E) Single‐cell RNA‐seq data (GSE202710) were analysed. (C) UMAP visualisation of cell clusters in murine bone marrow based on gene expression profiles. (D) Cell–cell interaction analysis of the Running group was performed. (E) GO analysis of genes upregulated in the Running group, with terms related to mitochondria visualised. (F) HIS‐SIM images of RAW264.7 cells co‐cultured with BMSCs, showing mitochondrial transfer from RAW264.7 cells (deep red) to BMSCs (green). Nuclei was stained with Hoechst (blue). (1) A field of view showing one BMSC and two RAW264.7 cells. Scale bar: 20 μm. (2) Magnified views of the boxed region in Panel 1. Scale bar: 5 μm. (3) Magnified view of the boxed region in Panel 2. Key frames were extracted from a 10‐min live‐cell imaging video, illustrating the process of RAW264.7 cell mitochondria integrating with BMSC mitochondria. Scale bar: 2 μm. (G, H) MTDR‐labelled RAW264.7 cells were treated with vehicle (Ctrl), Cytochalasin B (CytoB), GW4869 or a combination of CytoB and GW4869, then directly co‐cultured with MTG‐labelled BMSCs. Nuclei was stained with Hoechst (blue). Representative HIS‐SIM images (G) and quantification of mitochondrial transfer ratio (H) were shown. Two‐tailed unpaired Student's t‐test was performed. All error bars represent mean ± SD. *p < 0.05, **p < 0.01. ***p < 0.001.

To test our hypothesis regarding the role of mechanical tension in regulating macrophage mitochondrial function and its subsequent effects on BMSC osteogenesis, we first sought to establish an in vivo model that could effectively simulate mechanical loading conditions. Considering that running is a widely recognised model for simulating mechanical loading, we established a murine running model to investigate the effects of mechanical force on the osteoimmune microenvironment. Eight‐week‐old mice were subjected to 1 month of treadmill running before being sacrificed for analysis. Micro‐CT and HE staining revealed a significant increase in tibial trabecular bone mass (Figure 1A,B).

To further explore the effects of running on the bone marrow microenvironment, we identified and analysed publicly available single‐cell transcriptome data of mouse bone marrow from the GEO database (GSE202710), which includes samples from both running and control groups. Based on gene expression profiles, bone marrow cells were clustered into 12 groups, with macrophages ranking third and mesenchymal cells (BMSCs) ranking sixth (Figure 1C). Cell–cell communication analysis of the running group revealed strong interaction weights and strength between macrophages and BMSCs, suggesting that macrophages may regulate bone metabolism through interactions with BMSCs during running (Figure 1D). Considering that mitochondria play a central role in cellular energy metabolism and our study focuses on the metabolic response to mechanical stimulation, we specifically screened for differentially expressed mitochondria‐related genes within BMSCs and GO analysis of BMSCs revealed that these genes were significantly enriched in mitochondria‐related cellular component pathways, including mitochondrion, mitochondrion envelope, mitochondrion membrane and so forth (Figure 1E). These findings indicate that running likely impacts the structural and internal environment of BMSC mitochondria, thereby modulating their functional activities such as energy metabolism. Based on these observations, we hypothesised that interactions between macrophages and BMSCs during mechanotransduction might be closely associated with mitochondria.

To further validate this hypothesis, we established a direct co‐culture system comprising BMSCs and RAW264.7 cells. Prior to co‐culture, RAW264.7 cells were labelled with MitoTracker Deep Red (MTDR) to trace mitochondria, while BMSCs were labelled with MitoTracker Green (MTG). After 3 h of co‐culture, we conducted time lapse observations using live‐cell HIS‐SIM (video available in File S1). The recordings clearly demonstrated the transfer of mitochondria from RAW264.7 cells (deep red) to BMSCs (green) and their subsequent integration with BMSC mitochondria (Figure 1F). The directionality of mitochondrial transfer can be determined given the distinct size and shape difference between these two cell types. This finding confirms that under direct co‐culture conditions, macrophages can transfer mitochondria to BMSCs.

Mitochondrial transfer generally occurs via two mechanisms: direct cell‐to‐cell contact through TNTs or indirect transfer via extracellular vesicles (EVs) and free mitochondria [41]. To investigate the predominant route of mitochondrial transfer in our co‐culture system, RAW264.7 cells were treated with vehicle, the TNT formation inhibitor Cytochalasin B (CytoB), the EV release inhibitor GW4869 or a combination of both and then co‐cultured with BMSCs. HIS‐SIM imaging showed that inhibition of TNT formation led to a slight reduction in mitochondrial transfer, while inhibition of EV release resulted in a significant decrease. When TNT and EV pathways were simultaneously inhibited, mitochondrial transfer was nearly abolished (Figure 1G,H). These results suggest that both TNTs and EVs contribute to mitochondrial transfer from RAW264.7 cells to BMSCs, with EVs playing a more dominant role. However, to better mimic the in vivo microenvironment where cells interact without physical barriers, we adopted this direct co‐culture model for subsequent observations.

### 2.2 Mechanical Tension Altered Mitochondrial Dynamics of Macrophages

**FIGURE 2:**
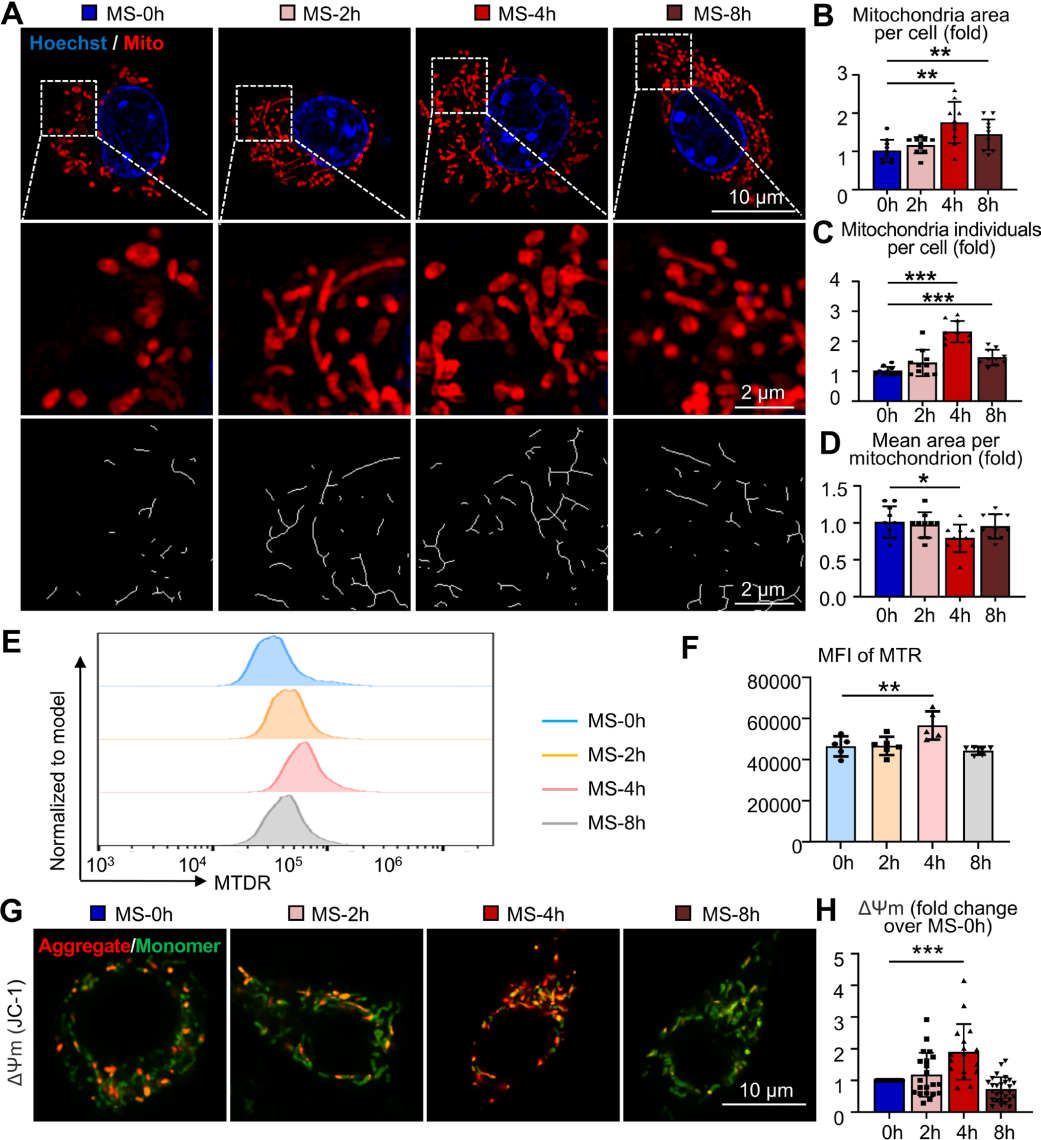
Mechanical tension altered mitochondrial dynamics of macrophages. (A–D) MTR (red) stained RAW264.7 cells were subjected to 0, 2, 4 and 8 h of mechanical tension and observed using HIS‐SIM. Nuclei was stained with Hoechst (blue). *N* = 10–15 cells per group. (A) Representative images. Top panel: full‐field images, scale bar: 10 μm. Middle panel: ×5 magnified view, scale bar: 2 μm. Bottom panel: simplified mitochondrial network morphology processed with ImageJ, Scale bar: 2 μm. (B–D) Quantification of fold changes in mitochondria area per cell (B), mitochondria number per cell (C) and mean area per mitochondrion (D). (E, F) MTR‐stained RAW264.7 cells were subjected to 0, 2, 4 and 8 of mechanical tension and analysed via flow cytometry. Representative histograms (E) and quantification of the MFI of MTR (F) were shown. (G, H) ΔΨ_m_ was assessed by JC‐1 probe and observed under super‐resolution microscopy. Representative images (G) and quantification of relative ΔΨ_m_ (H) were shown. Relative ΔΨ_m_ was analysed by calculating the aggregate (red)/monomer (green) ratio. Scale bar: 10 μm. One‐way ANOVA followed by Dunnett's post hoc multiple comparisons was performed. All error bars represent mean ± SD. *p < 0.05, **p < 0.01. ***p < 0.001.

Having confirmed that macrophages can transfer mitochondria to BMSCs under direct co‐culture conditions, we next sought to investigate how macrophages themselves respond to mechanical stimulation. To simulate the mechanical environment in vivo, we utilised a Flexcell FX‐5000 Tension system for applying mechanical stimulation to macrophages. Specifically, RAW264.7 cells were seeded onto collagen‐coated BioFlex six‐well plates and subjected to cyclic mechanical stretching (MS) for a duration of 0, 2, 4 and 8 h (MS‐0, MS‐2, MS‐4, MS‐8 h). To assess mitochondrial changes, RAW264.7 cells were labelled with MitoTracker Red CMXRos (MTR) and observed using HIS‐SIM (Figure 2A). Mitochondrial morphology and quantity were analysed using the MiNA macro‐tool in ImageJ. Notably, in the MS‐4 h group, macrophages exhibited an increase in mitochondrial number and a decrease in average mitochondrial size (Figure 2B–D), suggesting mitochondrial fission. To further confirm this observation, we measured the mean fluorescence intensity (MFI) of MTR via flow cytometry. Consistently, the MS‐4 h group showed a significant increase in MFI (Figure 2E,F), indicating an increase in mitochondrial quantity.

Previous studies have demonstrated that mitochondrial morphology affects its function [33]. Therefore, we hypothesised that mechanical stretch‐induced changes in macrophage mitochondrial morphology may also alter mitochondrial function. Mitochondrial membrane potential (ΔΨ_m_), a key indicator of mitochondrial function, regulates inner membrane permeability, thus impacting metabolism and energy production. We utilised the JC‐1 probe to assess mitochondrial membrane potential. Confocal results demonstrated that, consistent with previous findings, the mitochondrial membrane potential of RAW264.7 cells increased following 4 h of mechanical stretch (Figure 2G,H). This increase suggests an enhanced proton gradient, potentially reflecting higher energy accumulation in mitochondria. Collectively, these results indicate that 4 h of mechanical stretch alters macrophage mitochondrial dynamics, leading to increased mitochondrial quantity, a shift towards fission and enhanced energy metabolism.

### 2.3 Mechanical Tension Promotes Mitochondrial Transfer From Macrophages to BMSCs In Vitro and In Vivo

**FIGURE 3:**
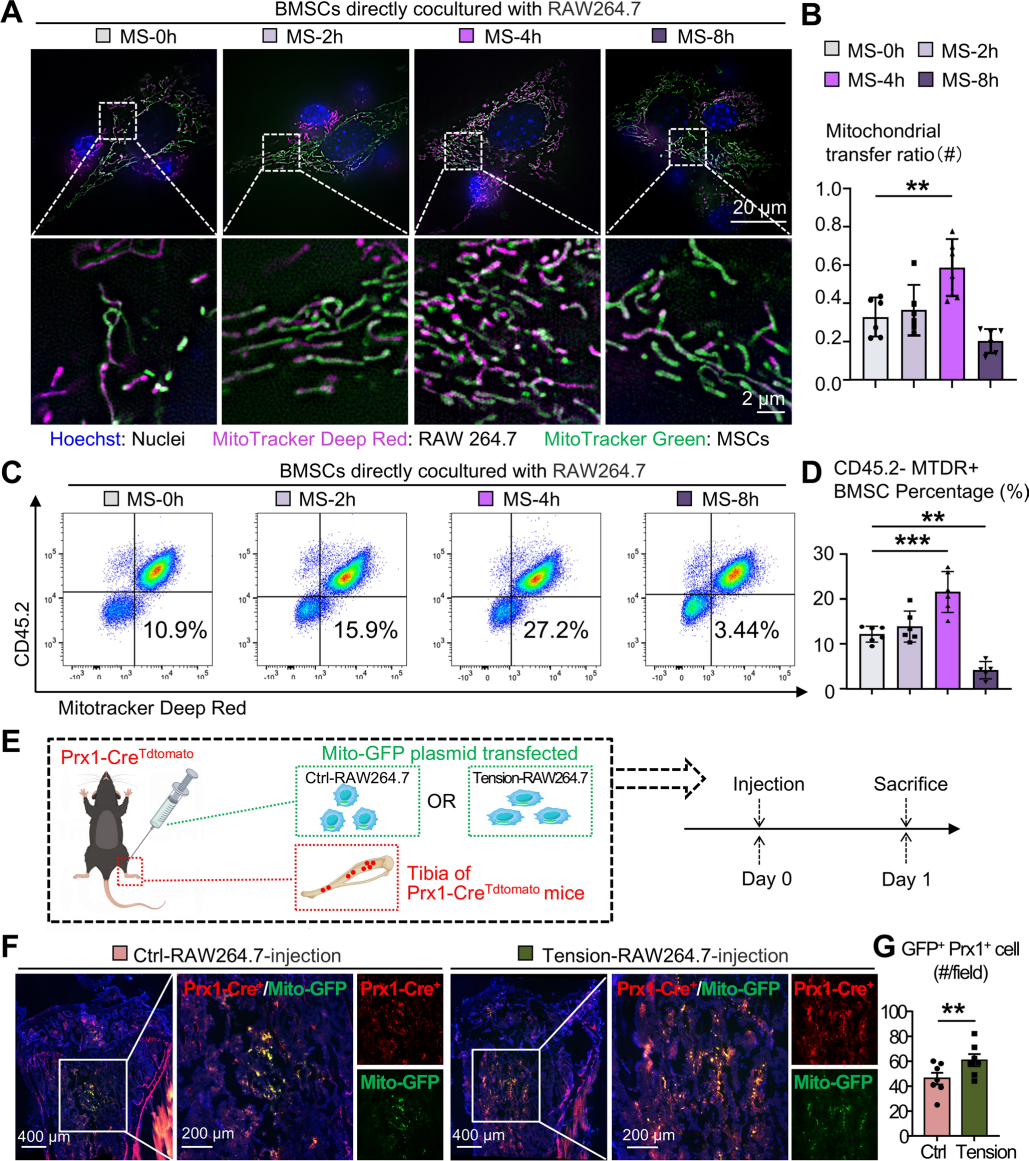
Mechanical tension promotes mitochondria transfer from Macrophage to BMSC in vitro and in vivo. (A, B) RAW264.7 cells subjected to 0, 2, 4 and 8 h of mechanical stretch were directly co‐cultured with BMSCs for 3 h and observed using HIS‐SIM. (A) Representative images showing mitochondrial transfer from macrophages (deep red) to MSCs (green). Nuclei was stained with Hoechst (blue). Top panel: scale bar, 20 μm. Bottom panel: zoomed views of the regions within the white dashed boxes in the top panel. Scale bar, 2 μm. (B) Quantification of RAW264.7 mitochondrial transfer ratio. *N* = 6 per group. (C, D) MTDR‐stained RAW264.7 cells subjected to 0, 2, 4 and 8 of mechanical stretch were directly co‐cultured with BMSCs for 3 h and analysed via flow cytometry. Representative scatter plots (C) and quantification (D) of the percentage of CD45.2^−^MTDR^+^ BMSCs among total cells. (E) Schematic of the experimental design: RAW264.7 cells transfected with pAcGFP1‐Mito plasmid were injected into the tibial bone marrow cavity of Prx1‐Cre^+^Tdtomato mice, and the mice were sacrificed 24 h post‐injection. (F, G) Tibial frozen sections from Prx1‐Cre^+^Tdtomato mice were stained with DAPI and observed under a fluorescence microscope. *N* = 6 per group. (F) Representative images showing mitochondrial transfer from injected RAW264.7 cells (GFP, green) to Prx1^+^ MSCs (Tdtomato, red). Scale bar, left panel: 400 μm; right panel: 200 μm. (G) Quantification of Prx1^+^GFP^+^ double‐positive cells. Two‐tailed unpaired Student's *t* test was performed. *p < 0.05. Two‐tailed unpaired Student's *t* test was performed for *F*. One‐way ANOVA followed by Dunnett's post hoc multiple comparisons was performed for all the others. All error bars represent mean ± SD. *p < 0.05, **p < 0.01. ***p < 0.001.

Having demonstrated that mechanical tension alters mitochondrial dynamics and enhances mitochondrial function in macrophages, we next sought to determine whether these changes also influence the mitochondrial transfer process observed in vitro (Figure 1H). Similarly, we subjected RAW264.7 cells to 0, 2, 4 and 8 h of mechanical stretch. RAW264.7 cells were labelled with MTDR, while BMSC were labelled with MTG. After this, RAW264.7 cells were co‐cultured with BMSCs for 3 h and then observed. The confocal images indicated that RAW264.7 cells subjected to 4 h of mechanical stretch transferred the most mitochondria to BMSCs, as observed by the MTDR‐labelled mitochondria integrating with the MTG‐labelled mitochondria in BMSCs (Figure 3A,B).

To further validate these findings and rule out the possibility of reverse mitochondrial transfer, flow cytometry was conducted. RAW264.7 cells, pre‐labelled with MTDR and subjected to the same durations of mechanical stretch, were co‐cultured with BMSCs for 3 h before flow cytometry analysis. CD45.2 was used to distinguish RAW264.7 cells (CD45.2^+^) from BMSCs (CD45.2^−^). The proportion of MTDR^+^ cells within the CD45.2^−^ BMSCs population was quantified as the mitochondrial transfer rate. Consistent with our microscopy observations, RAW264.7 cells subjected to 4 h of mechanical stretch demonstrated the highest mitochondrial transfer rate (Figure 3C,D). These results collectively suggest that 4 h of mechanical stretch optimally promotes mitochondrial transfer from macrophages to BMSCs in vitro. Accordingly, we will use this 4‐h duration as the mechanical stimulation condition in subsequent experiments.

To investigate the in vivo effect of mechanical tension on mitochondrial transfer from macrophages to BMSCs, we generated Prx1‐Cre^tdTomato^ mice, in which Prx1‐expressing MSCs are specifically labelled with tdTomato fluorescence. Simultaneously, we constructed a pAcGFP1‐Mito plasmid to fluorescently label the mitochondria of RAW264.7 cells. RAW264.7 cells transfected with the pAcGFP1‐Mito plasmid were subjected to 4 h of mechanical stretch or maintained as untreated controls, harvested and subsequently injected into the tibial bone marrow cavities of mice. The mice were sacrificed 24 h post‐injection for analysis (Figure 3E). Immunofluorescence staining of 8 μm‐thick frozen sections, representing a single‐cell layer, revealed co‐localisation of Prx1^+^ MSCs and RAW264.7 cell‐derived mitochondria in both groups (Figure 3F). Notably, the number of co‐localised cells was significantly higher in the group injected with tension stimulated RAW264.7 cells (Figure 3G). These findings indicate that mechanically stretched macrophages can transfer more mitochondria to BMSCs in vivo.

### 2.4 Mechanical Tension Promotes Drp1 Expression and Mitochondrial Localization in Macrophages

**FIGURE 4:**
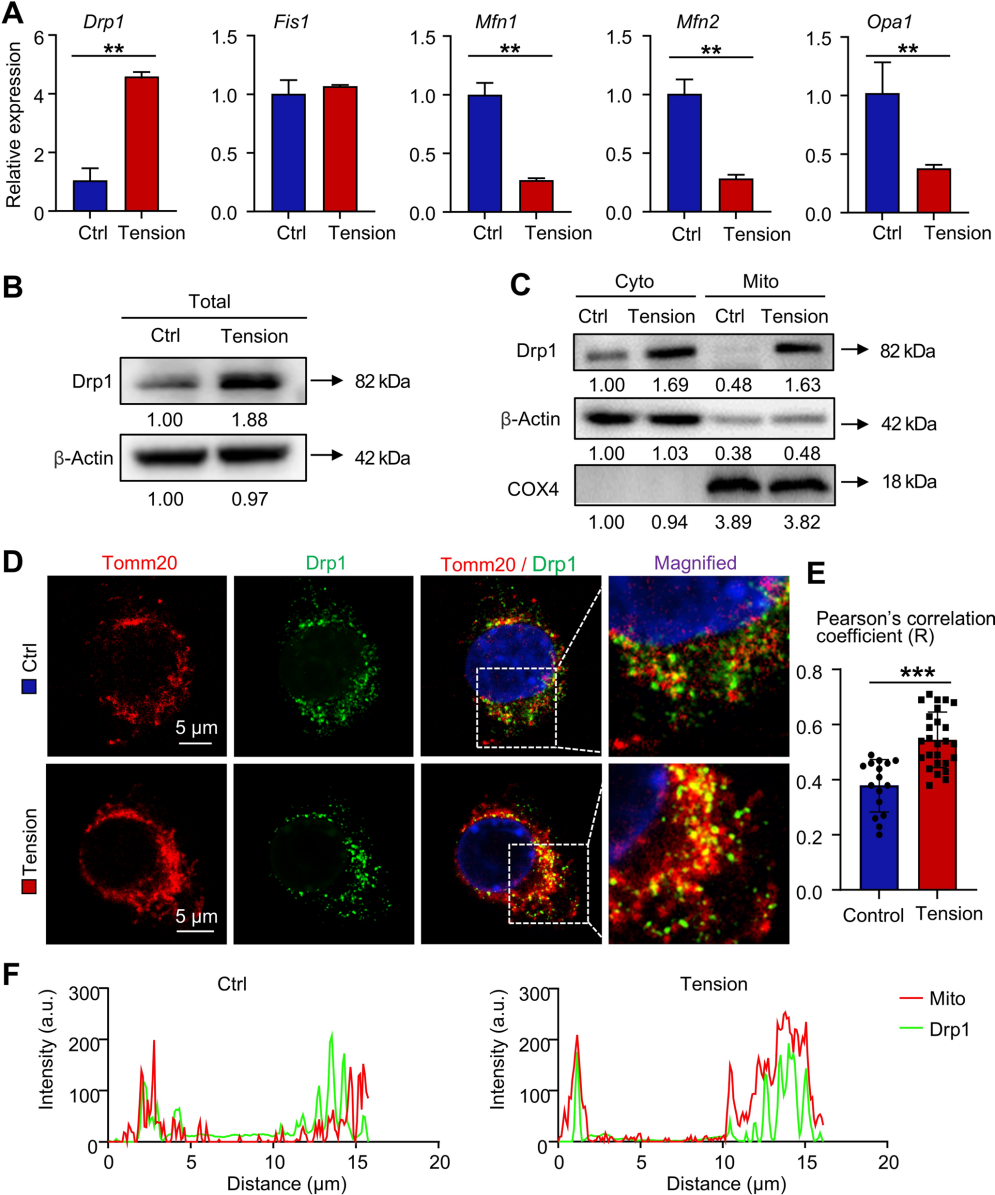
Mechanical tension increases Drp1 expression in mitochondria in macrophages. (A) Relative gene expression of *Drp1*, *Fis1*, *Mfn1*, *Mfn2* and *Opa1* in RAW264.7 cells from the Control and Tension groups measured by RT‐qPCR. Two‐tailed unpaired Student's *t* test was performed. (B) Western blot analysis of Drp1 expression levels in total cell lysates of RAW264.7 cells from the Control and Tension groups, normalised to β‐actin. (C) Western blot analysis of Drp1 expression levels in the cytoplasmic and mitochondrial proteins of RAW264.7 cells from the Control and Tension groups, normalised to β‐actin and COX4, respectively. (D, E) RAW264.7 cells from the Control and Tension groups were subjected to immunofluorescence staining for Tomm20 (red) and Drp1 (green) and mounted with DAPI. Representative images under confocal microscopy (D), quantification of Pearson's correlation coefficient (E) and quantitative colocalization profiles (F). Scale bar: 5 μm. Two‐tailed unpaired Student's t‐test was performed. All error bars represent mean ± SD. *p < 0.05, **p < 0.01. ***p < 0.001.

Having established that mechanical tension enhances mitochondrial transfer from macrophages to BMSCs both in vitro and in vivo, we next aimed to elucidate the underlying molecular mechanisms by which mechanical stimulation modulates mitochondrial dynamics in macrophages. Mitochondrial dynamics, characterised by a continuous balance between fusion and fission, are tightly regulated by specific proteins. Mitochondrial fission is predominantly mediated by Drp1 and Fis1, while fusion is primarily regulated by outer membrane proteins Mfn1 and Mfn2, as well as the inner membrane protein OPA1 [33]. To investigate the molecular mechanisms behind mitochondrial morphology and function changes in macrophages under mechanical stimulation, we exposed RAW264.7 cells to 4 h of mechanical tension (hereafter referred to as the Tension group). Cells cultured under identical conditions without mechanical tension were designated as the Control group. Using qPCR, we analysed the relative mRNA expression levels of genes encoding these proteins. Compared to the Control group, the Tension group exhibited a significant upregulation of the fission‐related gene *Drp1*, while the expression of *Fis1* remained unchanged. Conversely, the fusion‐related genes *Mfn1*, *Mfn2* and *Opa1* showed significant downregulation (Figure 4A). Consistent with the qPCR findings, western blot analysis indicated a significant increase in Drp1 protein expression in the Tension group (Figure 4B). Taken together, these results suggest that mechanical force‐induced mitochondrial fission is primarily associated with Drp1 regulation.

To elucidate the specific role of Drp1, we fractionated the total protein into cytoplasmic and mitochondrial components. We found that Drp1 expression was upregulated in both the cytoplasm and mitochondria of the Tension group, with a particularly notable increase in the mitochondria (Figure 4C). Since Drp1 is synthesised in the cytoplasm, this observation suggests that mechanical tension not only elevates Drp1 expression but also induces its translocation from the cytoplasm to mitochondria, consistent with Drp1's established role in mediating mitochondrial fission. To further validate these findings, immunofluorescence staining was performed. The confocal microscopy results showed increased co‐localization of Tomm20^+^ mitochondria with Drp1 in RAW264.7 cells from the Tension group (Figure 4D). The Pearson co‐localization coefficient and quantitative colocalization profiles further corroborated the mitochondrial enrichment of Drp1 under mechanical stimulation (Figure 4E,F). Collectively, these results demonstrate that mechanical tension upregulates Drp1 expression in macrophages and facilitates its translocation from the cytoplasm to the mitochondria.

### 2.5 Drp1 Upregulates Mitochondria Fission, Promoting Mitochondria Transfer From Macrophages to BMSCs

**FIGURE 5:**
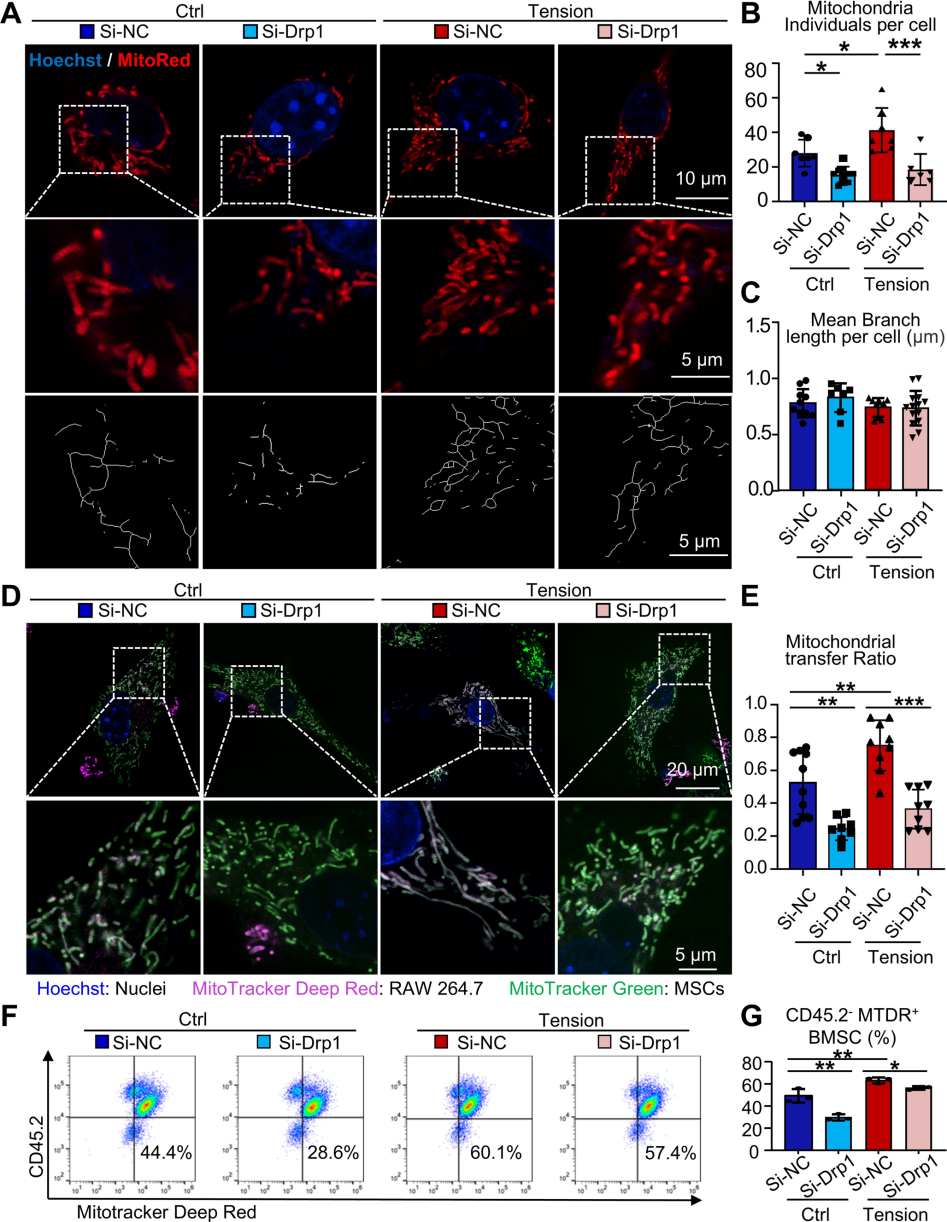
Drp1 upregulates mitochondria fission, promoting mitochondria transfer from macrophage to BMSC. (A–C) MTR‐stained RAW264.7 cells from the Control and Tension groups with or without *Drp1* knockdown were observed using super‐resolution microscopy. (A) Top panel: Representative images. Scale bar, 10 μm; Middle panel: Zoomed‐in view within the white dashed box, Scale bar, 5 μm; Bottom panel: Simplified mitochondrial morphology via ImageJ, Scale bar, 5 μm. (B) Quantification of mitochondria individuals per cell. (C) Quantification of mean branch length per cell. (D, E) RAW264.7 cells from the Control and Tension groups with or without Drp1 knockdown were directly co‐cultured with BMSCs for 3 h and observed using super‐resolution microscopy. (D) Representative images showing mitochondrial transfer from injected RAW264.7 cells (deep red) to BMSCs (green). (E) Quantification of mitochondrial transfer ratio. Top panel: Representative images. Scale bar, 20 μm; Bottom panel: Zoomed‐in view within the white dashed box, Scale bar, 5 μm. (F, G) RAW264.7 cells from the Control and Tension groups with or without *Drp1* knockdown were directly co‐cultured with BMSCs for 3 h and analysed via flow cytometry. Representative scatter plots (F) and quantification of CD45.2^−^MTDR^+^ MSCs percentage in total cells (G). Two‐tailed unpaired Student's *t* test was performed for G within the Tension group. One‐way ANOVA followed by Dunnett's post hoc multiple comparisons was performed for all the others. All error bars represent mean ± SD. *p < 0.05, **p < 0.01. ***p < 0.001.

Building upon our findings that mechanical tension induces Drp1 upregulation and mitochondrial translocation in macrophages, we further investigated whether Drp1‐mediated mitochondrial fission directly contributes to the enhanced mitochondrial transfer from macrophages to BMSCs. To this end, we employed siRNA to knock down *Drp1* expression in both the Control and Tension groups, creating four experimental groups: Control‐Si‐NC, Control‐Si‐Drp1, Tension‐Si‐NC and Tension‐Si‐Drp1. Mitochondria were labelled with MTR, and mitochondrial morphology in RAW264.7 cells was observed (Figure 5A). The confocal microscopy results demonstrate that mitochondrial fission induced by mechanical tension can be mitigated by Drp1 knockdown, as indicated by a significant decrease in the average number of mitochondria per cell (Figure 5B). However, no significant changes were observed in the average mitochondrial branch length across the four groups (Figure 5C).

Furthermore, to investigate whether the changes in mitochondrial morphology in macrophages under mechanical tension are related to mitochondrial transfer, the four groups of MTDR‐pre‐labelled macrophages were co‐cultured with MTG‐pre‐labelled BMSCs. After 3 h of co‐culture, mitochondrial transfer was observed using confocal microscopy (Figure 5D). In both the Control and Tension groups, Drp1 knockdown reduced the proportion of mitochondrial transfer (Figure 5E), indicating the mechanical tension‐induced promotion of mitochondrial transfer was reversed by Drp1 knockdown. To further confirm these findings, flow cytometry was conducted. RAW264.7 cells from each of the four groups were pre‐labelled with MTDR and co‐cultured with BMSCs for 3 h before flow cytometry analysis. CD45.2 was used to distinguish RAW264.7 cells (CD45.2^+^) from BMSCs (CD45.2^−^) (Figure 5F). The proportion of MTDR^+^ cells in the CD45.2‐BMSCs population was defined as the mitochondrial transfer rate. Consistent with our microscopy observations, Drp1 knockdown led to a relative reduction in mitochondrial transfer rate in both the Control and Tension groups. These findings suggest that mechanical tension increases mitochondrial number and promotes Drp1‐mediated mitochondrial transfer from macrophages to BMSCs.

### 2.6 Mito‐EVs From Mechanical‐Stretched Macrophages Promote BMSCs Osteogenetic Differentiation

**FIGURE 6:**
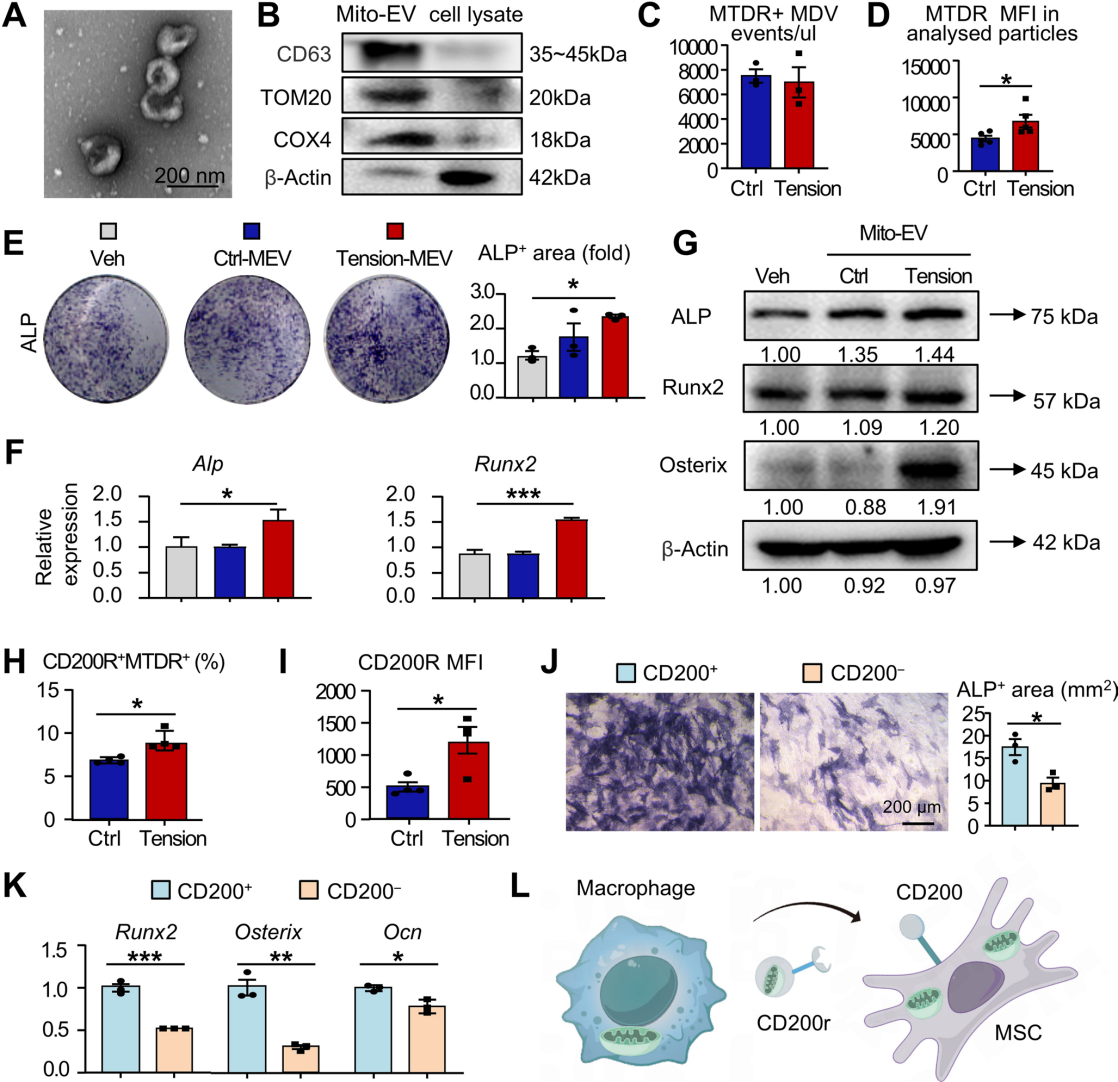
Mito‐EVs from mechanical‐stretched macrophages promote BMSCs osteogenetic differentiation through CD200R‐ ligand pathway. (A) Transmission electron microscopy image of Mito‐EV morphology. Scale bar: 200 nm. (B) Western blot analysis of CD63, TOM20, COX4 and β‐Actin expression in Mito‐EVs and cell lysates of RAW264.7 cells. (C, D) Flow cytometry analysis of Mito‐EVs from Control and Tension groups. Quantification of MTDR^+^ Mito‐EV events (C) and MTDR MFI in analysed particles (D). *N* = 3 per group. (E) ALP staining and quantification of ALP^+^ area. (F) Relative gene expression of *Alp* and *Runx2* in BMSCs treated with no Mito‐EVs, Control Mito‐EVs or Tension Mito‐EVs using RT‐qPCR. (G) Western blot analysis of ALP, Runx2 and Osterix protein levels in BMSCs, normalised to β‐Actin. (H, I) Flow cytometry analysis of Mito‐EVs from Control and Tension groups. Quantification of the proportion of CD200R^+^ MTDR^+^ Mito‐EVs (I) and MFI of CD200R (J). *N* = 4 per group. (J, K) CD200^+^ and CD200^−^ MSCs were sorted by flow cytometry. (J) ALP staining after 7 days of osteogenic induction with quantification of ALP^+^ area. Scale bar,100 μm. (K) Relative gene expression of *Runx2*, *Osterix* and *Ocn* in MSCs after 7 days of osteogenic induction by RT‐qPCR. (L) Schematic: MSCs recognise Mito‐EVs secreted by macrophages via the CD200R‐CD200 interaction. Two‐tailed unpaired Student's *t* test was performed. All error bars represent mean ± SD. *p < 0.05, **p < 0.01. ***p < 0.001.

Having demonstrated that mechanical tension enhances Drp1‐mediated mitochondrial transfer from macrophages to BMSCs, we next explored the functional impact of these transferred mitochondria. To this end, we isolated Mito‐EVs from the culture medium of RAW264.7 cells following a differential centrifugation protocol as previously described [43]. Transmission electron microscopy (TEM) showed that isolated vesicles had sphere‐shaped morphology (Figure 6A), consistent with previously reported [44]. Western blot analysis further confirmed the presence of the exosomal marker CD63, as well as the mitochondrial outer membrane marker Tomm20 and the inner membrane marker COX4 (Figure 6B). These findings indicate that the isolated vesicles possess characteristic features of exosomes while also carrying mitochondrial components, supporting their classification as Mito‐EVs.

Given our prior observation of increased mitochondrial count in macrophages under mechanical tension, we hypothesised that Mito‐EVs secretion might also increase as a mechanism to maintain mitochondrial homeostasis. To evaluate this, RAW264.7 cells were pre‐labelled with MTDR and subjected to either control or mechanical tension conditions. Quantification of Mito‐EVs was performed using bead‐assisted flow cytometry, as previously reported [45]. The analysis revealed no significant difference in the quantity of Mito‐EVs secretion between the two groups (Figure 6C). However, the MFI of MTDR per particle was significantly higher in the Tension group (Figure 6D), suggesting an increased mitochondrial content within each Mito‐EV under mechanical tension.

To examine the impact of Mito‐EVs on BMSCs osteogenic differentiation under mechanical tension, BMSCs were subjected to osteogenic induction culture with the addition of Mito‐EVs isolated from cells under no tension (referred to as Ctrl‐MEV group) and mechanical tension (referred to as Tension‐MEV group), while a group without Mito‐EV addition served as the vehicle. After 7 days of osteogenic induction, ALP staining revealed a significant increase in the ALP‐positive area in the Tension‐MEV group compared to the Vehicle group (Figure 6E). RT‐qPCR analysis demonstrated significantly upregulated mRNA expression levels of *Alp* and *Runx2* in the Tension‐MEV group compared to the other two groups (Figure 6F). Western blot results further confirmed that the expression of ALP, Runx2 and Osterix was significantly elevated in the Tension‐MEV group compared to the Ctrl‐MEV and Vehicle groups (Figure 6G). These findings collectively indicate that Tension‐MEV plays a critical role in promoting the osteogenic differentiation of BMSCs.

Exogenous mitochondria are known to enhance BMSC osteogenesis through metabolic regulation; however, the mechanism by which Mito‐EVs exert their effects remains unclear [46, 47]. Previous studies have shown that macrophage‐derived Mito‐EVs express CD200R [43]. Additionally, its ligand, CD200, was identified as a potential new MSC marker [48]. CD200^+^ MSCs have also been reported to generate ectopic bone in nude mice [48] and exhibit higher osteogenic differentiation ability [49]. Considering this background, we hypothesise that the CD200R‐CD200 interaction may be involved in the regulatory effect of Mito‐EVs on BMSCs. Flow cytometry analysis revealed that macrophage‐derived Mito‐EVs express CD200R (Figure 6H), while BMSCs express CD200 [49, 50]. In the Tension group, the proportion of CD200R^+^ Mito‐EVs significantly increased (Figure 6H), accompanied by elevated CD200R expression per EV (Figure 6I). To investigate the role of CD200, primary MSCs were sorted into CD200^+^ and CD200^−^ populations by flow cytometry and subjected to osteogenic induction [48]. CD200^+^ MSCs exhibited significantly larger ALP^+^ areas and higher expression of osteogenic genes, including *Runx2*, *Osterix* and *Ocn*, compared to CD200^−^ MSCs (Figure 6J,K). These findings suggest that CD200^+^ MSCs possess greater osteogenic potential. Combined with the observed upregulation of CD200R expression in Tension‐MEVs, we propose that the enhanced osteogenic effects of Tension‐MEVs on BMSCs may be partially attributed to the interaction between CD200R on Mito‐EVs and CD200 on MSCs, facilitating efficient mitochondrial transfer and uptake (Figure 6L).

### 2.7 Mitochondria From Mechanical‐Stretched Macrophages Promote Bone Formation Through the CD200R‐CD200 Interaction In Vivo

**FIGURE 7:**
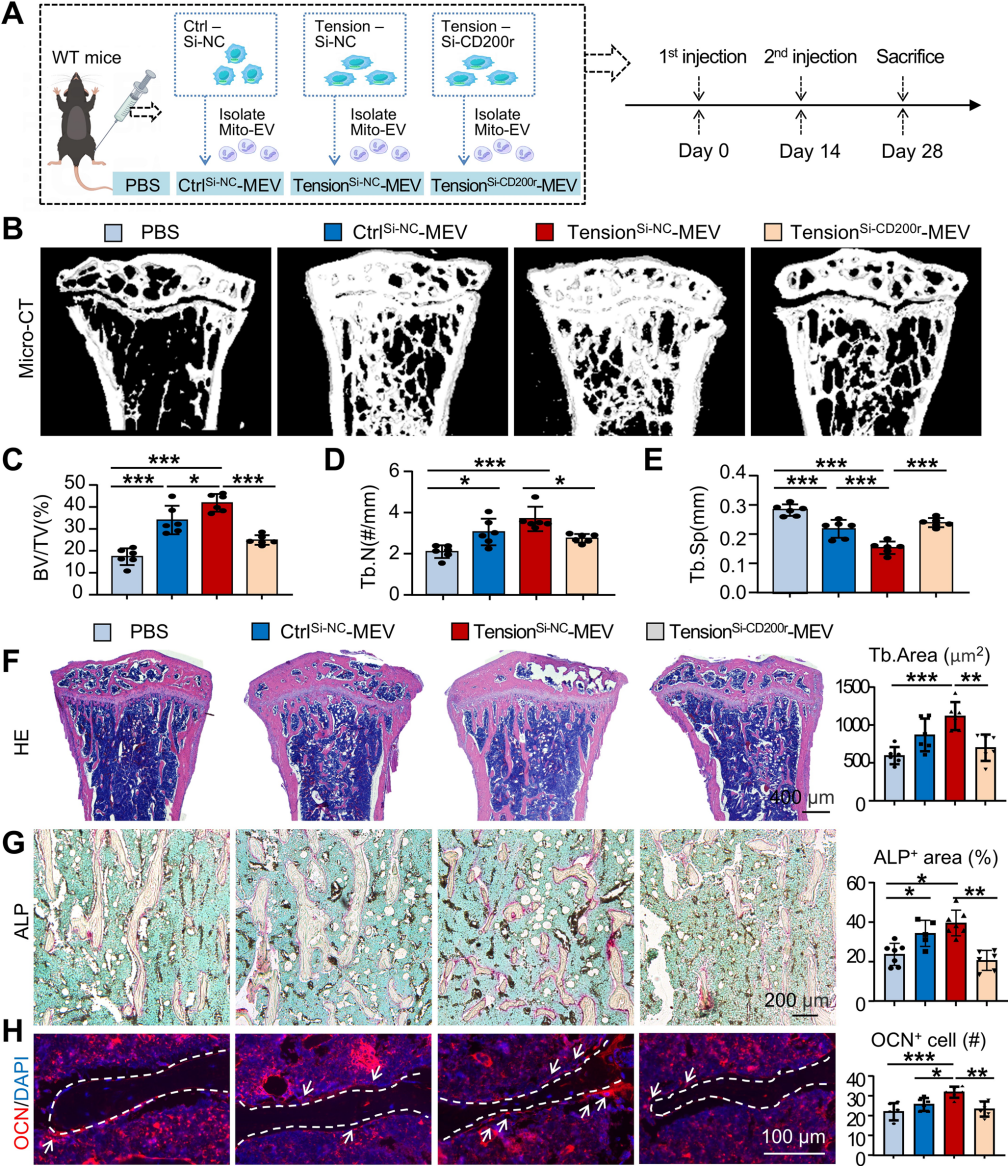
Mitochondria from mechanical‐stretched macrophages promote bone formation in vivo. (A–H) Eight‐week‐old mice were injected into the tibial bone marrow cavity with PBS, Ctrl^Si‐NC^‐MEV, Tension^Si‐NC^‐MEV or Tension^Si‐CD200r^‐MEV every 2 weeks for a total of 1 month, followed by sacrifice and analysis (*N* = 6 per group). (A) Experimental design. (B) Representative micro‐CT reconstructed images of the tibia. (C–E) BV/TV (%) (E), Tb. N (#/mm) (F) and Tb. Sp (mm) (G) was determined. (F) Representative HE‐stained paraffin sections of the tibia. Scale bar: 400 μm. Trabecular bone area (μm^2^/field) in the tibia was determined. (G) Representative ALP‐stained paraffin sections of the tibia. Scale bar: 200 μm. ALP^+^ area (%) in the tibia was determined. (H) Representative OCN immunofluorescence‐stained paraffin sections of the tibia. Scale bar: 100 μm. OCN^+^ cell number around the trabecular bone surface (#/field) was determined. The region within the white dashed line represents the trabecular bone area. White arrows indicate representative OCN^+^ cells. One‐way ANOVA followed by Dunnett's post hoc multiple comparisons was performed, *N* = 6 per group. All error bars represent mean ± SD. *p < 0.05, **p < 0.01. ***p < 0.001.

Building upon the in vitro evidence that Mito‐EVs derived from mechanically‐stretched macrophages enhance BMSC osteogenesis potentially via the CD200R‐CD200 signalling axis, we further investigated the in vivo effects of these Mito‐EVs on bone formation and examined the functional significance of the CD200R‐CD200 interaction using a murine Mito‐EV injection model. We isolated Mito‐EVs from RAW264.7 cells across three groups: the non‐tensioned control group (Ctrl^Si‐NC^‐MEV), the tensioned group (Tension^Si‐NC^‐MEV) and the tensioned group with CD200R knocked down using siRNA (Tension^SiCD200r^‐MEV). These Mito‐EVs were then locally injected into the tibial bone marrow cavity of mice every 2 weeks for 1 month, with a PBS‐injected group serving as vehicle control (Figure 7A).

Micro‐CT analysis revealed that compared to the PBS‐injected group, both Ctrl^Si‐NC^‐MEV and Tension^Si‐NC^‐MEV injections promoted an increase in trabecular bone mass in the tibia. Among these, the increase in bone mass was most significant in the Tension‐MEV group (Figure 7B), as demonstrated by the increased bone volume/total volume (BV/TV), trabecular number (Tb. N), as well as the decreased trabecular separation (Tb.Sp). In contrast, mice injected with Tension^SiCD200r^‐MEV showed no significant changes in these variables (Figure 7C–E). HE‐staining showed a significant increase in trabecular bone area in mice injected with Ctrl^Si‐NC^‐MEV and Tension^Si‐NC^‐MEV, with the most pronounced increase observed in the Tension^Si‐NC^‐MEV group, while no significant changes were observed in the Tension^SiCD200r^‐MEV group (Figure 7F). Similarly, ALP staining revealed a marked increase in ALP‐positive osteoblast area in the Ctrl^Si‐NC^‐MEV and Tension^Si‐NC^‐MEV groups, with the Tension^Si‐NC^‐MEV group exhibiting the most substantial increase. Again, the Tension^SiCD200r^‐MEV group exhibited no significant changes (Figure 7G). Similarly, immunofluorescence staining for OCN showed a significant increase in OCN^+^ osteoblasts in the Ctrl^Si‐NC^‐MEV and Tension^Si‐NC^‐MEV groups, with the highest increase observed in the Tension‐MEV group, whereas no significant changes were observed in the Tension^SiCD200r^‐MEV group (Figure 7H). These results demonstrate that mitochondria from mechanically stretched macrophages enhance the osteogenic activity of BMSCs and promote bone formation in vivo. The CD200R‐CD200 interaction likely facilitates the recognition and uptake of mitochondria transferred through EVs.

## 3 Discussion

Mechanical stimuli are widely recognised for their influence on bone biology, as they play a crucial role in regulating bone remodelling and maintaining bone homeostasis. Numerous studies have demonstrated that mechanical stimulation modulates the bone immune microenvironment, thereby promoting bone formation and remodelling [21, 35]. In addition, bone formation is highly dependent on mitochondrial energy supply. Correspondingly, a recent study has found that piezoelectric stimulation may enhance the osteogenic differentiation of stem cells by regulating mitochondrial ATP metabolism [51]. Based on these findings, we hypothesis

e that mitochondria may play a role in the regulation of the osteoimmune microenvironment under mechanical stimulation. Here, we established an in vitro MS model and discovered that cyclic tension induces mitochondrial fission in macrophages. As most research on the relationship between mechanical tension and mitochondrial dynamics focused on cell types like fibroblasts and epithelial cells [37, 38], our study provides a novel perspective for macrophages in this context. However, the correlation between mitochondrial morphology and cellular metabolic state varies across different cell types [52, 53], which requires further investigation.

Since most of the mitochondrial fission events require Drp1, we explored its role in mechanically induced mitochondrial fission. Drp1, a GTPase belonging to the dynamin superfamily, is recruited from the cytoplasm to the mitochondrial outer membrane by receptor proteins in response to specific signals, where it mediates membrane constriction via GTP hydrolysis to promote mitochondrial fission [54]. Fis1 is one of the known Drp1 receptors and is typically involved in coordinating mitochondrial fission. However, in our study, despite the upregulation of Drp1, Fis1 expression remained unchanged, prompting our reflection. To date, the mechanisms of mitochondrial fission remain incompletely understood. A recent study has identified two spatially distinct modes of fission: midzone division, occurring at the centre of mitochondria and associated with MFF (another receptor of Drp1) and endoplasmic reticulum contact [55]; and peripheral division, which takes place at the periphery of mitochondria and is linked to Fis1 and lysosomal contact [56]. This may explain why some studies have found that Fis1 depletion has minimal effects on mitochondrial fission [54, 57]. Additionally, post‐translational modifications of Drp1, other receptor proteins such as MID51 and MID49 and even cell‐type‐specific differences may also influence mitochondrial fission [54]. Future studies are required to explore the distinct mechanisms.

Mitochondrial transfer has been observed between various cell types [41], including a recent study reporting its occurrence from macrophages to BMSCs [39]. Using super‐resolution fluorescence microscopy, we confirmed this process and found that macrophages subjected to mechanical tension for 4 h—coinciding with maximal morphological changes—transferred the highest amounts of mitochondria to BMSCs. This raised the question of whether mitochondrial morphology might influence the transfer rate. Previous research suggests that M2 macrophages, which exhibit more fragmented mitochondria than M1 or M0 types, transfer more mitochondria to MDA‐MB‐231 cells, implying that smaller mitochondrial structures may facilitate transfer [27]. In this study, we reached a similar conclusion that mitochondrial fission promotes the transfer of mitochondria from macrophages to BMSCs.

Given that mitochondria were predominantly transferred via EVs under our co‐culture conditions, our study primarily focused on the effects of EV‐mediated mitochondrial transfer on BMSCs. Mito‐EVs are a type of EV containing mitochondrial components, which are highly heterogeneous in size, density and surface markers [58]. For instance, a recent study isolated a novel Mito‐EV subpopulation from the brain, termed mitovesicles (approximately 50–200 nm), which were enriched in mitochondrial components but lacked conventional EV markers [59]. Another study identified large EVs (> 1 μm in diameter) containing intact mitochondria [60]. Here, we adopted a conventional differential centrifugation‐based method [61]. However, due to the lack of specific isolation methods for Mito‐EVs, this method may also capture unwanted particles and other contamination. In the future, immune‐isolation approaches based on certain mitochondrial proteins could be explored to further purify Mito‐EVs.

It has been demonstrated that internalised mitochondria enhance the osteogenic differentiation of BMSCs through classical energy metabolism pathways [46]. In this study, we validated a novel immunoregulatory pathway via Mito‐EVs through both in vivo and in vitro experiments. Previous research has reported that the CD200R‐CD200 interaction facilitates EV‐mediated mitochondrial transfer from macrophages to sensory neurons [43]. Additionally, CD200 has been identified as a potential marker of MSCs [48]. Thus, we hypothesise that macrophage‐derived Mito‐EVs can be recognised and internalised by MSCs through CD200R‐CD200 binding, facilitating their docking to BMSCs. Through dual mechanisms of metabolic and immune modulation, exogenous mitochondrial transfer and the CD200R‐CD200 interaction may work synergistically to promote BMSCs osteogenesis.

However, we acknowledge certain limitations in our in vivo experimental design. Specifically, we did not observe direct evidence that mechanical forces (e.g., running) promote increased mitochondrial transfer from macrophages to BMSCs in vivo. Additionally, we were unable to validate the role of the CD200R‐CD200 axis in mitochondrial transfer using in vivo approaches. These limitations stem from the current lack of techniques to endogenously label macrophage mitochondria. Moreover, RAW264.7 cells, although widely used and offering significant advantages in experimental reproducibility and consistency [62, 63], may not fully replicate physiological conditions. In future studies, our laboratory will generate LysM^Cre^‐MitoDendra2^flox^ mice and utilise primary macrophages to better validate our conclusions. In addition, screening experiments should be conducted to further explore other potentially involved signalling pathways beyond the CD200R–CD200 axis. Notably, under mechanical force conditions, the possibility that macrophages transfer mitochondria to other osteogenic lineage cells, such as osteoblasts and osteocytes, cannot be excluded. On the other hand, considering the crucial role of mitochondria in promoting osteoclast activities [64], it is possible that macrophages, after donating mitochondria, may experience reduced proliferation and differentiation, potentially leading to impaired osteoclast formation and decreased bone resorption, which represents another promising research direction.

## 4 Conclusions

**FIGURE 8:**
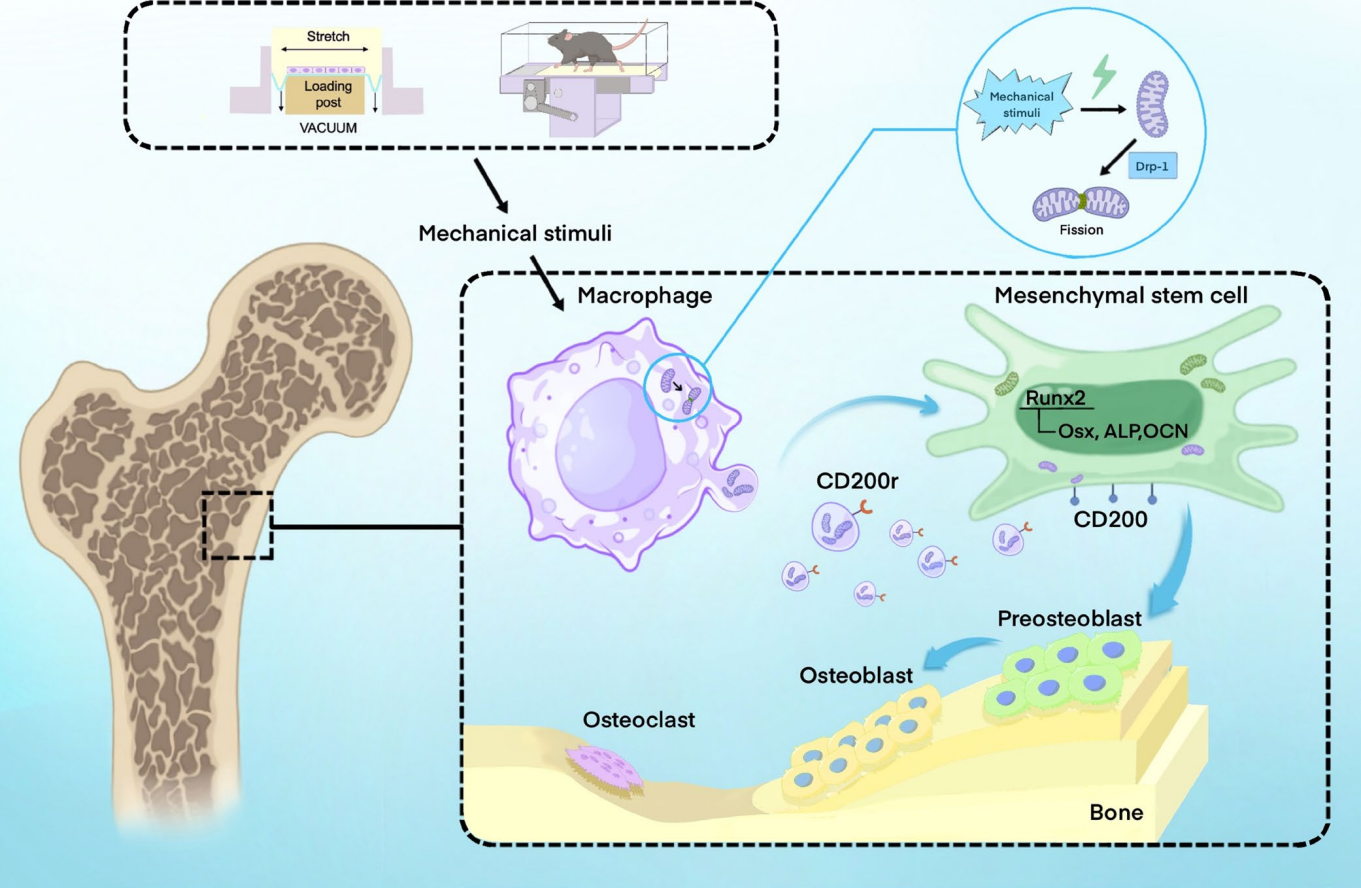
Schematic representation. Mechanical stimulation promotes mitochondrial fission in macrophages, a process mediated by Drp1. The fragmented mitochondria are partly secreted extracellularly in the form of Mito‐EVs and transferred to MSCs via the CD200R‐CD200 interaction. This process enhances the expression of osteogenesis‐related genes such as *Runx2*, *Osx* and *ALP* in MSCs, ultimately promoting osteogenic differentiation of MSCs and bone formation.

In summary, our study reveals that mechanical tension activates Drp1‐mediated mitochondrial fission in macrophages, facilitating the release of Mito‐EVs that are subsequently transferred to BMSCs. Additionally, the CD200R‐CD200 interaction enhances the uptake of these mechanically stimulated macrophage‐derived Mito‐EVs by BMSCs, ultimately promoting osteogenic differentiation (Figure 8). Collectively, our research elucidates how mechanical force regulates the osteoimmune microenvironment through mitochondrial dynamics, providing valuable insights for future theoretical studies and potential clinical applications of mitochondrial‐based therapies in bone repair and regeneration.

## 5 Methods

### 5.1 Animals

Eight‐week‐old male C57BL/6 wild‐type mice and Prx1‐Cre^tdTomato^ mice were utilised in this study. Prx1‐Cre^tdTomato^ mice were obtained by crossing Prx1‐Cre male mice with ROSA^tdTomato^ female mice. All mice were bred at the SPF‐grade Animal Experimental Center of Nanjing Medical University. The animal experiments were approved by the IACUC of Nanjing Medical University (Approval No. 2308033).

1. For treadmill running, mice were subjected to a daily exercise regimen for 30 min. During the first 10 min, the mice ran at a speed of 6 m/min with an acceleration of 1 m/s. For the remaining 20 min, the running speed was maintained at 12 m/min. The exercise protocol was conducted 5 days per week for 4 consecutive weeks. Following the exercise period, the mice were sacrificed and tissue samples were collected for further analysis.
2. To validate mitochondrial transfer in vivo, the tibial bone marrow cavity of the mice was locally injected with approximately 6 × 10^5^ pAcGFP1‐Mito plasmid‐transfected RAW264.7 cells using a Bioflex plate. Mice were euthanised 24 h post‐injection.
3. For Mito‐EV therapy, 10 μL (2 μg/μL) of Mito‐EV was administered via local injection into the tibial bone marrow cavity every 2 weeks [22, 65]. Mice were euthanised 1 month post‐treatment.

### 5.2 Cell Culture

1. The mouse macrophage cell line RAW264.7 was obtained from the Cell Resource Center of the Shanghai Institutes for Biological Sciences, Chinese Academy of Sciences. Cells were cultured in DMEM supplemented with 10% foetal bovine serum and 1% penicillin–streptomycin (P/S).
2. BMSCs were isolated from the tibiae and femora of 3‐ to 4‐week‐old pathogen‐free C57BL/6 mice, following our previously reported protocol [66]. Briefly, mice were euthanised, long bones were dissected, rinsed with PBS and cut into small pieces. These bone fragments were cultured in plastic dishes containing α‐MEM with 10% FBS and 1% P/S for 3 days. Afterward, the bone pieces were transferred to clean dishes for a second passage and cultured for an additional 7 days to allow for cell growth and confluence. Cells were passaged at a 1:2 ratio when they reached 80% confluence. Third passage BMSCs were utilised for subsequent experiments.
3. For co‐culture of RAW264.7 and BMSCs, 1 × 10^5^ BMSCs were seeded in confocal glass‐bottom dishes (Cellvis, 35 mm) for 24 h. RAW264.7 cells, with or without treatment, were then detached using PBS and seeded at a density of 4 × 10^5^ cells onto the BMSCs.

All cells were maintained at 37°C in a 5% CO_2_ atmosphere.

### 5.3 Drug Treatment of RAW264.7 Macrophages

To inhibit TNT formation, RAW264.7 cells were pretreated with fresh culture medium containing 1 μM CytoB (MCE) for 6 h. To inhibit EV release, RAW264.7 cells were pretreated with fresh culture medium containing 20 μM GW4869 (MCE) for 6 h. All procedures were performed according to the manufacturers' instructions. Cells were then subjected to subsequent experiments.

### 5.4 Flexcell Strain Unit Application

Flexcell FX‐5000 Tension system (Flexcell International Corporation, USA) is a computer‐controlled system that allows for the application of programmable mechanical strain to cultured cells. This system provides precise control over strain magnitude, frequency and duration and is used extensively in mechanobiological studies [67, 68].

To apply mechanical stimulation, RAW264.7 cells were uniformly seeded in collagen I‐coated six‐well BioFlex culture plates with a flexible silicone membrane (Flexcell International Corporation, USA). After adhesion, RAW264.7 cells were exposed to Flexcell cyclic sinusoidal continuous stretch strain (10%, 0.5 Hz) for 0, 2, 4 and 8 h under 37°C in a 5% CO_2_ atmosphere. Cells maintained in the same culture plates but not subjected to stretching served as controls.

### 5.5 Small‐Interfering RNA (siRNA) and Plasmid Transfection

For the transfection of RAW264.7 cells with siRNA, we constructed Si‐Drp1 (Corues Biotech, China) and Si‐CD200r1 (Corues Biotech, China). A total of 50 nM siRNA was used and subsequent co‐culture experiments were conducted 24 h post‐transfection. For plasmid transfection in RAW264.7 cells, we utilised the pAcGFP1‐Mito plasmid (Corues Biotech, China), with subsequent cell stretching experiments performed 24 h after transfection. Lipofectamine 2000 (Invitrogen) was employed as the transfection reagent and all transfection protocols were carried out in accordance with the manufacturer's guidelines.

### 5.6 Live Cell Mitochondrial Staining

Mitochondria were labelled using MTR (C1035, Beyotime, China), MTG (C1048, Beyotime, China) and MTDR FM (C1032, Beyotime, China). Cell nuclei were stained with Hoechst 33342 live cell staining solution (100×) (C1029, Beyotime, China). All staining procedures followed the manufacturer's instructions. Briefly, a pre‐warmed working solution of 100 nM MitoTracker was co‐incubated with adherent cells for15–30 min. The Hoechst 33342 staining solution (10 μL per 1 mL of culture medium) was applied for 10 min, followed by three washes with PBS and replacement with fresh culture medium. Specifically, after removing the Mito‐Tracker working solution, cells were washed three times with PBS and incubated in complete medium for 1 h to ensure the removal of unbound dye before proceeding to co‐culture operations. For the assessment of mitochondrial membrane potential, a mitochondrial membrane potential detection kit (JC‐1, Beyotime, China) was utilised according to the manufacturer's instructions.

### 5.7 Super‐Resolution Fluorescence Imaging

Mitochondrial super‐resolution imaging was conducted using a commercially available High Intelligent and Sensitive Structured Illumination Microscope (HIS‐SIM) system provided by Guangzhou CSR Biotechnology Co. Ltd. Images were acquired with a 100×/1.5 NA oil immersion objective (Olympus). Cells were cultured in glass‐bottom dishes (35 mm, Cellvis) and maintained in a humidified chamber at 37°C with 5% CO_2_ for real‐time SIM imaging. SIM images were collected and analysed following previously established protocols [69]. Sparse deconvolution was performed to enhance image quality [70]. For mitochondrial morphology analysis, the MiNA macro‐tool in ImageJ (Media Cybernetics, USA) was utilised for semi‐automated analysis of mitochondrial network morphology, as previously described [71].

### 5.8 Cell Immunofluorescence

Cells were digested and cultured on chamber slides (Biologix, China). After attachment, cells were washed twice with PBS, fixed in 4% paraformaldehyde for 30 min and subsequently washed three times with PBS (5 min each). Samples were blocked with goat serum (BOSTER, China) in a 37°C incubator for 30 min. Subsequently, primary antibodies Tomm20 and Drp1 were applied and incubated overnight at 4°C. The next day, samples were washed three times with PBS. Species‐matched secondary antibodies were applied and incubated at 37°C for 1 h, followed by mounting with DAPI‐containing medium (VECTASHIELD with DAPI, Vectorlabs). Images were captured using a super‐resolution laser confocal microscope (AXE, Nikon). Quantitative colocalisation analysis was performed using the colocalisation plugin in ImageJ.

### 5.9 Flow Cytometry Analysis and Sorting

Quantitative assessment of mitochondrial transfer was performed as previously described [72]. Co‐cultured MTDR‐labelled RAW264.7 cells and BMSCs were digested with trypsin. Cells were then stained with FITC‐CD45.2 antibody (11‐0454‐85, eBioscience) at 4°C in the dark for 30 min, followed by analysis using a 23‐colour flow cytometer (FACSymphony A5, BD, USA). Results were analysed with FlowJo software (Version 10.8.1, FlowJo LLC, Ashland OR, USA). For the sorting of CD200, third‐passage MSCs were digested with trypsin and initially stained with Zombie Violet Fixable Viability Kit (423113, BioLegend) in the dark for 10 min to label dead cells. Subsequently, cells were stained with PE‐CD200 antibody (12‐5200‐80, Invitrogen) at 4°C in the dark for 30 min. Analysis was performed using a 15‐colour cell sorter (FACSAria Fusion, BD, USA) and results were processed with FlowJo software (Version 10.8.1, FlowJo LLC, Ashland OR, USA).

### 5.10 Extraction, Morphological Characterisation and Flow Cytometric Analysis of Mito‐EVs

The extraction of EVs was based on a previously reported method [61]. Briefly, 1 × 10^6^ RAW264.7 cells were cultured in each well of a Bioflex six‐well plate. The cells were grown to 80% confluency, washed with PBS and then cultured in the exosome‐free medium for 24 h before conditioned medium (CM) collection. The CM was centrifuged at 300*g* for 10 min, followed by 2000g for 10 min and then 10,000*g* for 30 min to remove cells and debris. The resulting supernatant was then ultracentrifuged at 100,000*g* for 70 min. The pellet was washed with PBS and subjected to a second ultracentrifugation at 100,000*g* for 70 min to ensure purity. The extracted EVs were resuspended in PBS and stored at −80°C for further analysis.

1. For morphological characterisation, 3 μL of the extracted EV solution was placed on a copper‐coated grid and negatively stained with 2% phosphotungstic acid for 10 min. After drying under a bright light for 2 min, imaging was performed using a transmission electron microscope (JEOL, Japan).
2. For flow cytometric analysis of Mito‐EVs, a CytoFLEX flow cytometer (CytoFLEX 5, Beckman Coulter, USA) was employed according to a previously reported method [45]. RAW264.7 cells, either with or without stimulation, were pre‐stained with MTDR and EVs were extracted after 24 h as described above. For exosome quantification, the analysis was conducted at an appropriate dilution factor, using an equal number of particles or volume as the endpoint condition. PE‐CD200 antibody was incubated with EVs in the dark for 15 min before detection. Results were analysed using Cytexpert 2.5 software (Beckman Coulter, USA).

### 5.11 Alkaline Phosphatase Staining

For the detection of alkaline phosphatase (ALP) activity, BMSCs were cultured in osteogenic induction medium (α‐MEM containing 10% foetal bovine serum, 50 μg/mL ascorbic acid and 10 mM β‐glycerophosphate) for 7 days, with or without the addition of extracted Mito‐EV (50 μg/mL) [22, 73]. Samples were stained according to the manufacturer's instructions using the BCIP/NBT alkaline phosphatase colour development kit (Beyotime, China). All images were captured using a GE Image Scanner III and staining was observed under a microscope. Quantification was conducted using ImageJ software in three randomly selected regions.

### 5.12 Real‐Time Quantitative PCR (RT‐qPCR)

Total RNA was extracted using TRIzol Reagent (Invitrogen, Carlsbad, CA, USA). cDNA synthesis was performed with PrimeScript RT Master Mix (Cat#RR036A, Takara) and quantified via RT‐qPCR using specific primers. The primer sequences are detailed in Table S1. Quantitative PCR was performed using the ABI QuantStudio 7 Real‐Time PCR System (Applied Biosystems, USA). Gene expression normalisation was achieved using the ΔΔ*C*_t_ method, which calculates relative quantities (RQ) or fold changes by comparing the cycle threshold (*C*_t_) values of the target gene against those of a housekeeping gene. The formula applied is as follows: RQ = 2^−ΔΔ*C*t^, where ΔΔ*C*_t_ = Δ*C*_t_ (target gene) − Δ*C*_t_ (housekeeping gene).

### 5.13 Western Blot

Whole‐cell lysates were prepared by direct lysis in RIPA buffer. For mitochondrial protein isolation, a mitochondrial separation kit (C3601, Beyotime, China) was employed, following the manufacturer's protocol to obtain mitochondrial protein samples, while the remaining supernatant contained cytoplasmic proteins devoid of mitochondria. Protein concentrations were determined using a BCA protein assay kit (Beyotime, Cat#P0012), followed by separation via SDS‐PAGE and transfer to a polyvinylidene fluoride (PVDF) membrane. The membrane was blocked in PBS containing 5% non‐fat dry milk for 3 h at room temperature. Subsequently, the membrane was incubated overnight at 4°C with the primary antibody. The following day, it was washed three times with PBST (10 min each) and incubated with an HRP‐conjugated secondary antibody for 1 h. Enhanced chemiluminescence (ECL) reagent (Tanon) was used for detection and visualisation was performed using the Tanon‐5200 multichannel chemiluminescence system (Tanon).

### 5.14 Micro‐CT and Histology

Tibial samples were extracted and fixed in 4% paraformaldehyde solution, followed by scanning with a high‐resolution micro‐CT (Skyscan 1176, Bruker, Germany) at a resolution of 15.6 μm. Three‐dimensional images were reconstructed for analysis (CTAn, Skyscan), focusing on cross‐sectional images of the distal tibia. Based on previously reported regions of interest (VOI), volume measurements were conducted. The proximal metaphysis was designated as a target area of 2 mm, starting from the distal end of the proximal growth plate and extending distally. Bone volume fraction (BV/TV), trabecular thickness (Tb.Th), trabecular number (Tb.N) and separation (Tb.Sp) were calculated with values set between 50 and 255.

1. For frozen sections, tibial tissue samples from Prx1‐Cre^tdTomato^ mice were fixed in 4% paraformaldehyde, decalcified in 14% EDTA, dehydrated in 30% sucrose and embedded in OCT compound (Sakura). Sections of 8 μm were cut using a rotary microtome (Leica, Wetzlar, Germany) and examined using an inverted fluorescence microscope (Leica, Germany) for fluorescence imaging.
2. For paraffin sections, tibial tissue samples from WT mice were fixed in 4% paraformaldehyde, decalcified in 14% EDTA, dehydrated and embedded in paraffin. Sections of 4 μm were obtained using a rotary microtome (Leica, Wetzlar, Germany). HE and ALP staining were performed on paraffin sections for subsequent analysis as previously described.

### 5.15 Bioinformatics Analysis

The dataset used in this study was obtained from the Gene Expression Omnibus (GEO) public database (GSE202710, available at: https://www.ncbi.nlm.nih.gov/geo/query/acc.cgi?acc=GSE202710).

1. For the processing of the raw data, we used the R package Seurat (version 5.1.0) to process the unique molecular identifier (UMI) count matrix. The following cells were filtered out: (1) those with fewer than 200 genes, fewer than 1000 UMIs or log10 GenesPerUMI less than 0.7; (2) cells where mitochondrial gene counts exceeded 10% or cells with more than 5% of their count attributed to haemoglobin genes. Data normalisation was performed using the NormalizeData function in Seurat. We selected variable genes using the FindVariableFeatures function (using the Variance Stabilising Transformation method). Clustering of cells based on gene expression profiles was performed using the FindClusters function. UMAP visualisation was used to display the cells. We identified marker genes for each cluster using the FindAllMarkers function (test.use = wilcox). The marker genes are shown in the table celltype_markers (File S2). Clustering based on typical gene markers is shown in the marker gene violin plots (Figure S1).
2. For the intercellular communication analysis, we used the mouse version of the CellChat database and extracted the ‘Secreted Signalling’ pathways for subsequent analysis. High‐expression genes and interactions were identified using the identifyOverExpressedGenes and identifyOverExpressedInteractions functions. After projecting gene data onto the PPI (protein–protein interaction) network, we calculated communication probabilities and retained communications involving at least 10 cells. The intensity of interactions between different cell types was visualised using the netVisual_circle function.
3. For the Gene Ontology (GO) analysis, we performed differential expression analysis between two groups using the FindMarkers function to identify genes that were significantly different between the two groups. We then used the subset function to further filter the significant marker genes with adjusted *p*‐values (p_val_adj) less than 0.05 and avg_log2FC greater than 0.5. The gene symbols were converted to ENTREZIDs using the bitr function and the data were merged into a new data frame. GO analysis was performed on the top 5000 genes with elevated expression in the running group using g:Profiler (https://biit.cs.ut.ee/gprofiler/gost) and mitochondrial‐related pathways were visualised using the ggplot function.

### 5.16 Statistical Analysis

All results are given as mean ± SD. Statistical analysis was performed using GraphPad Prism 7 software (GraphPad Software Inc., San Diego, CA, USA). Comparisons between two groups were analysed using the two‐tailed unpaired Student's *t* test. Comparisons among three or more groups were carried out using one‐way ANOVA followed by Dunnett's post hoc multiple comparisons test. *p* Values less than 0.05 were considered statistically significant. All experiments were performed in three independent biological replicates.

## Author Contributions

Y.L., Z.Y., Y.C., H.C., Y.C., Y.D. and R.J. organised the samples, conducted the experiments and performed data analysis. Y.C. and W.S. provided experimental and technical support. Y.L., W.S. and H.W. prepared the manuscript. H.W. designed the experiments, interpreted the data and revised the manuscript.

## Ethics Statement

All animal experimental procedures were conducted according to guidelines approved by the Animal Care Committee of Nanjing Medical University (No. 2308033).

## Consent

All authors agree to be published.

## Conflicts of Interest

The authors declare no conflicts of interest.

## Supporting information


**File S1:** An accelerated time‐lapse video of a continuous 10‐min recording demonstrates mitochondrial transfer from RAW264.7 cells to BMSCs under direct co‐culture conditions. The mitochondria of RAW264.7 cells were labelled with MTDR, while those of BMSCs were labelled with MTG. The video was captured using HIS‐SIM.

**File S2:** We identified marker genes for each cluster using the FindAllMarkers function (test.use = wilcox). The marker genes are shown.

**Data S1:** Supporting Information.

## Data Availability

The data that support the findings of this study are available from the corresponding author upon reasonable request.
