# Supplementary material for: Mechanical Force Promotes Mitochondrial Transfer From Macrophages to BMSCs to Enhance Bone Formation
Source: Cell Prolif. 2025 Aug 27;59(5):e70121. doi: 10.1111/cpr.70121 (PMC13114792; doi:10.1111/cpr.70121)
Supplement: Supplementary file 3 — Data S1: Supporting Information. [file CPR-59-e70121-s002.docx]

**Supplemental Figure 1**

**
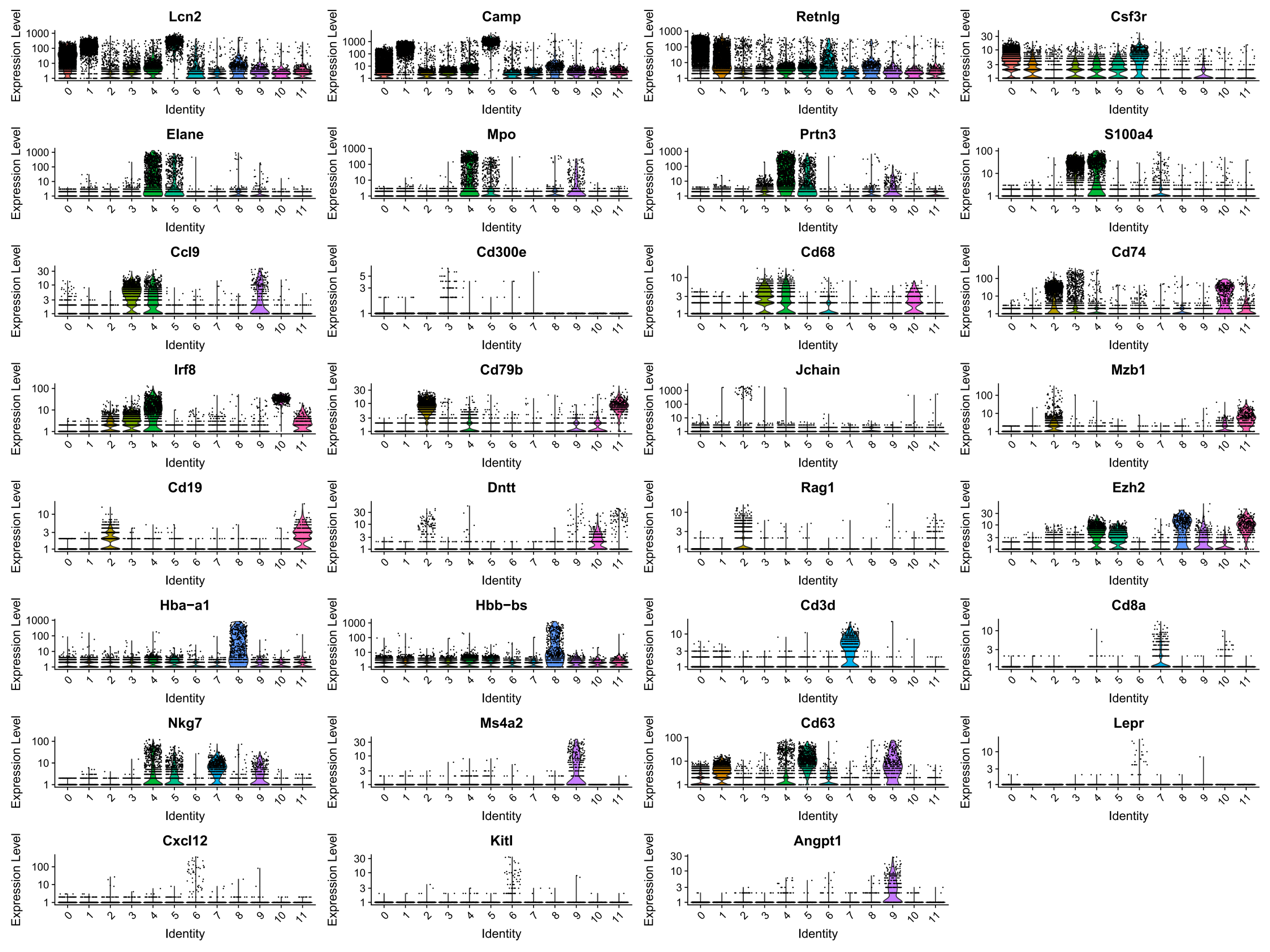
**We identified marker genes for each cluster using the FindAllMarkers function (test.use = wilcox). The marker genes are shown. Target cells were clustered based on typical marker genes. Violin plots of the marker genes are presented below.

| **Supplemental Table 1** | |  |
| --- | --- | --- |
| The sequences of primers used for RT-qPCR are listed below. | | |
| **Name** | **F/R** | **Sequences** |
| *mDrp1* | F | AGAAAATGGGGTGGAAGCAGA |
|  | R | AAGTGCCTCTGA TGTTGCCA |
| *mFis1* | F | CCGGCTCAAGGAATATGAAA |
|  | R | ACAGCCAGTCCAATGAGTCC |
| *mMfn1* | F | CCTACTGCTCCTTCTAACCCA |
|  | R | AGGGACGCCAATCCTGTGA |
| *mMfn2* | F | ATGTTACCACGGAGCTGGAC |
|  | R | AACTGCTTCTCCGTCTGCAT |
| *mOpa1* | F | ATACTGGGATCTGCTGTTGG |
|  | R | AAGTCAGGCACAATCCACTT |
| *mAlp* | F | CTTGCTGGTGGAAGGAGGCAGG |
|  | R | CACGTCTTCTCCACCGTGGGTC |
| *mRunx2* | F | ATGCTTCATTCGCCTCACAAA |
|  | R | GCACTCACTGACTCGGTTGG |
| *mCD200r1* | F | CCATCCTGCACAATAGCATACA |
|  | R | GGACAGAGATTGGTTACCAGTCA |
| *mGapdh* | F | GGTCGGTGTGAACGGATTTG |
|  | R | ATGAGCCCTTCCACAATG |
